# Histone Acetylation in Bone Remodeling: Metabolic Regulation, Lineage-Specific Chromatin Programs, and Therapeutic Implications

**DOI:** 10.3390/cells15211963

**Published:** 2026-10-28

**Authors:** Jianhong Dong, Xianji Wei, Yifan Qian, Yiming Qian, Wei Zhang

**Affiliations:** 1 Department of Pharmacy, Sir Run Run Shaw Hospital, School of Medicine, Zhejiang University, No. 3 East Qingchun Road, Hangzhou 310016, China; 2 College of Athletic Performance, Shanghai University of Sport, No. 399 Changhai Road, Yangpu District, Shanghai 200438, China; 3 Department of Pharmacy, Affiliated Hangzhou First People’s Hospital, School of Medicine, Westlake University, No. 261 Huansha Road, Shangcheng District, Hangzhou 310006, China; 4 School of Exercise and Health, Shanghai University of Sport, No. 399 Changhai Road, Yangpu District, Shanghai 200438, China

**Keywords:** bone remodeling, histone acetylation, acetyl-CoA compartmentalization, acetyl-CoA flux, remodeling-unit coupling

## Abstract

**Highlights:**

**What are the main findings?**

- Acetyl-CoA availability to chromatin, rather than total cellular levels, helps determine bone-cell transcriptional responses.
- These chromatin programs coordinate osteoblast, osteoclast, and osteocyte functions during bone remodeling.

**What are the implications of the main findings?**

- Bone epigenetic regulation should be interpreted beyond total acetyl-CoA or global histone acetylation.
- Therapeutic targeting will require cell- and context-specific strategies.

**Abstract:**

Bone remodeling requires coordinated transcriptional regulation across osteoblasts, osteoclasts, and osteocytes. Histone acetylation connects cellular metabolism to these lineage-specific programs, yet its regulatory effects depend not simply on total cellular acetyl-CoA abundance, but also on metabolic carbon routing, subcellular acetyl-CoA availability, and the targeting of chromatin-modifying enzymes to specific genomic regions. This review examines how these processes shape skeletal-cell differentiation, function, and communication within the bone-remodeling unit. We integrate evidence linking acetyl-CoA metabolism to histone acetylation across skeletal lineages, highlighting the distinct contributions of metabolic substrate utilization, acetylation and deacetylation machinery, and locus-specific chromatin regulation. We further discuss how mechanical loading, aging, inflammation, and metabolic disease reshape these regulatory networks and influence the balance between bone formation and resorption. Particular attention is given to the distinction between metabolic changes and their chromatin consequences, as well as to the experimental approaches needed to resolve acetyl-CoA compartmentalization and trace metabolic carbon into histone acetyl groups. Finally, we evaluate therapeutic opportunities and challenges in targeting metabolic–chromatin pathways while preserving physiological bone remodeling. Integrating metabolic routing, chromatin regulation, and intercellular communication provides a framework for understanding skeletal adaptation and identifying new avenues for therapeutic development.

## 1. Introduction

Bone remodeling maintains skeletal mass, microarchitecture, and mechanical competence through the coordinated activities of osteoblasts, osteoclasts, and osteocytes. RANKL–RANK, Wnt/β-catenin, BMP/SMAD, NF-κB, and NFATc1 signaling regulate lineage differentiation and remodeling activity, while cellular metabolism shapes the transcriptional responses to these signals. Metabolic flux therefore influences bone-cell function not only by supplying energy and biosynthetic substrates but also by regulating the metabolites available to chromatin-modifying enzymes and transcriptional regulators [1].

Histone acetylation provides a direct biochemical link between cellular metabolism and gene regulation. Lysine acetyltransferases use acetyl-CoA as an acetyl donor, whereas sirtuins require NAD^+^ for deacylation [2]. Glucose, fatty acids, acetate, and amino acids contribute to acetyl-CoA metabolism, but their contribution to histone acetylation depends on intracellular carbon routing and subcellular metabolite availability. In osteoclasts, ATP-citrate lyase (ACLY) and GCN5/KAT2A link citrate-derived acetyl-CoA to histone H3 acetylation and Rac1 transcription [3]. In bone marrow-derived mesenchymal stem/stromal cells (BMSCs), the mitochondrial citrate carrier (CiC/SLC25A1) supports carbon transfer to the nucleocytosolic compartment and osteogenic chromatin regulation [4,5]. Here, nucleocytosolic acetyl-CoA refers to acetyl-CoA in the nuclear and cytosolic compartments collectively, distinguishing it from total cellular acetyl-CoA without implying a discrete, isolated nuclear pool. Its availability for chromatin acetylation depends on metabolic production, transport, and consumption. Similarly, global histone acetylation reflects overall modification levels, whereas locus-specific measurements identify changes at defined genomic regulatory regions; chromatin accessibility provides complementary information about the regulatory state of chromatin.

The consequences of acetyl-CoA metabolism vary with skeletal lineage, differentiation stage, and genomic context. During Wnt3a-induced commitment of bipotent progenitors, reduced nuclear acetyl-CoA and global histone acetylation contribute to repression of the adipogenic program during early osteogenic specification [6]. Conversely, maintaining osteogenic chromatin in aged BMSCs and under ambient oxygen culture conditions depends on sufficient nucleocytosolic acetyl-CoA availability [4,5]. Acetyl-CoA metabolism also has distinct effects during osteoclast differentiation. ACLY-derived acetyl-CoA supports a pro-resorptive Rac1 transcriptional program, whereas acetate treatment during early differentiation restores H3K27ac and chromatin accessibility at IRF8-associated regulatory regions, suppressing osteoclastogenesis [7]. These findings illustrate how the metabolic context, developmental stage, and genomic targets of acetylation determine its functional consequences in skeletal cells.

**Figure 1:**
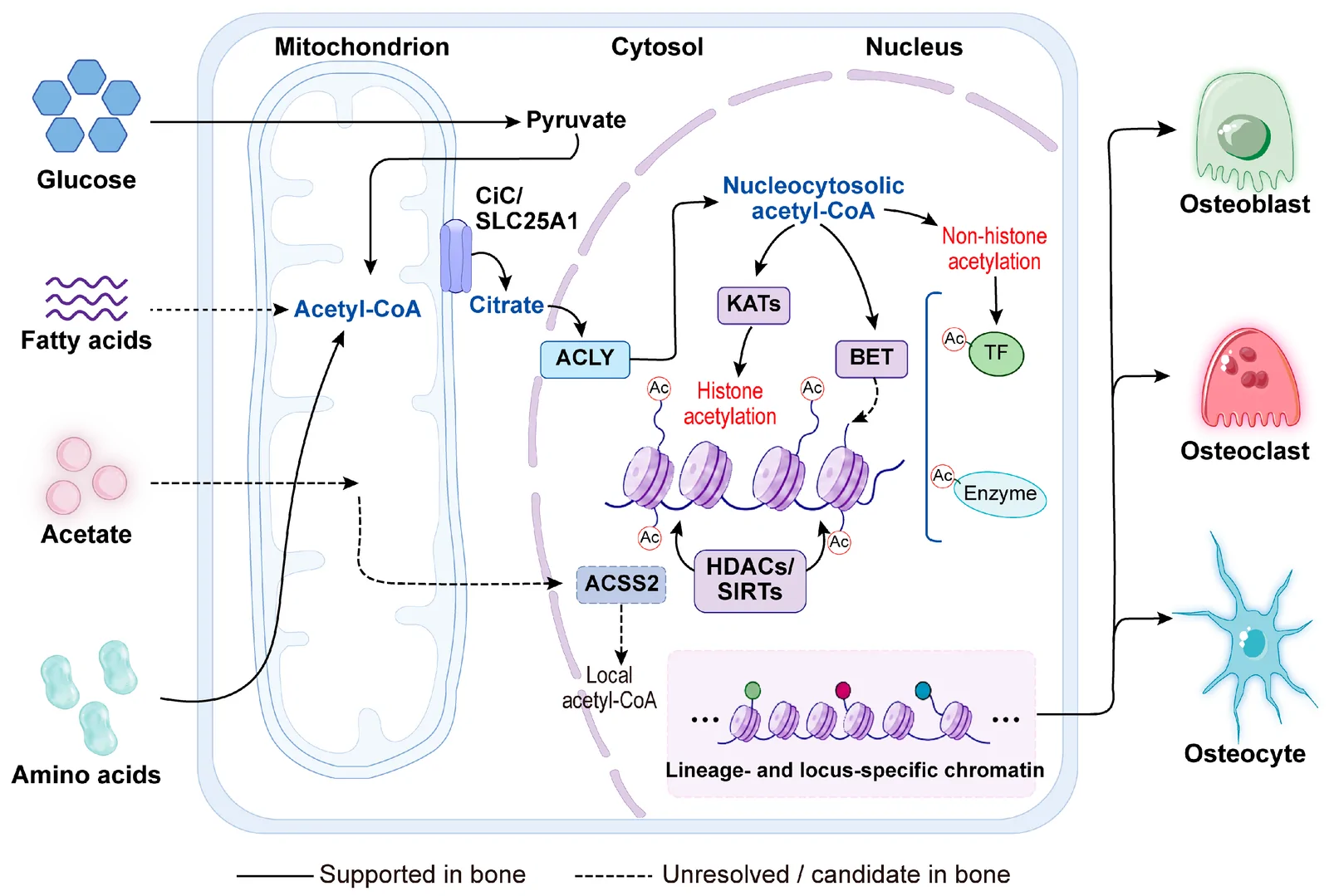
Evidence-guided framework of metabolic acetyl-CoA routing and histone acetylation in bone cells. Carbon derived from glucose, fatty acids, acetate, and amino acids contributes to acetyl-CoA metabolism through distinct intracellular pathways. Mitochondrial citrate is exported via CiC/SLC25A1 and cleaved by ACLY to generate nucleocytosolic acetyl-CoA. Lysine acetyltransferases (KATs), acetyl-lysine readers, and deacetylases regulate the establishment, recognition, and removal of histone acetylation marks. Lineage-specific transcription factors and genomic regulatory regions shape the resulting transcriptional programs in osteoblasts, osteoclasts, and osteocytes. Non-histone acetylation of transcription factors and metabolic proteins is depicted separately as a complementary regulatory mechanism. Solid arrows represent pathway steps supported by experimental evidence in skeletal cells, whereas dashed arrows indicate candidate or unresolved pathways, including mechanisms established in non-skeletal systems whose contributions to bone cells have not been demonstrated. Evidence supporting individual metabolic and chromatin steps does not necessarily establish direct incorporation of carbon from a particular substrate into histone acetyl groups. Representative experimental models and mechanistic evidence are summarized in Table 1.

This review examines histone acetylation in bone remodeling through an integrated framework linking metabolic carbon routing, compartment-specific acetyl-CoA availability, and lineage-specific chromatin regulation (Figure 1). We first consider how metabolic pathways determine acetyl-CoA availability, followed by the chromatin machinery that interprets these metabolic inputs in distinct skeletal lineages and differentiation states. We then examine how these mechanisms integrate within the bone-remodeling unit and how aging, inflammation, mechanical loading, and metabolic disease reshape skeletal adaptation. Finally, we discuss therapeutic opportunities and the challenges of targeting metabolic–chromatin pathways while preserving coordinated bone formation and resorption.

The primary focus is the metabolic regulation of histone acetylation and associated chromatin accessibility in skeletal cells. Non-histone acetylation is considered where it provides complementary insight into acetyl-CoA metabolism, transcriptional regulation, or communication within the skeletal microenvironment. Table 1 summarizes representative metabolic–chromatin pathways alongside their experimental models, chromatin readouts, and supporting evidence, allowing direct comparison of mechanisms established in skeletal systems with complementary findings from non-skeletal models.

This narrative review draws on English-language literature identified through searches of PubMed and Web of Science, covering publications from January 2010 to the most recent literature search. Search terms addressed bone remodeling, osteoblasts, osteoclasts, osteocytes, histone acetylation, chromatin regulation, acetyl-CoA metabolism, and related metabolic and epigenetic regulators. Selection prioritized original experimental studies linking metabolic perturbations to histone or chromatin changes and skeletal-cell function. Relevant studies from non-skeletal systems were included to inform mechanisms and methodologies that complement the skeletal literature, together with selected foundational publications predating 2010.

## 2. Metabolic Routing and Compartment-Specific Acetyl-CoA Accessibility in Bone Cells

The contribution of metabolism to histone acetylation depends not only on carbon source but also on intracellular routing and the availability of acetyl-CoA to chromatin-modifying enzymes. This section examines citrate-dependent acetyl-CoA regulation in osteoclasts and osteoblast-lineage cells, followed by acetate-sensitive chromatin responses and the contributions of glucose and fatty-acid metabolism. These pathways differ in their experimental support, but together they illustrate how metabolic routing shapes transcriptional programs across skeletal lineages and differentiation stages.

### 2.1. Acetyl-CoA Regulation Across Osteoclast Differentiation and Resorptive Function

During RANKL-induced osteoclastogenesis, ATP-citrate lyase (ACLY) generates nucleocytosolic acetyl-CoA from mitochondria-derived citrate and supports histone H3 acetylation. Genetic depletion or pharmacological inhibition of ACLY reduces acetyl-CoA availability, histone H3 acetylation, multinucleated osteoclast formation, and bone resorption. GCN5/KAT2A acts downstream of ACLY, and its overexpression partially restores Rac1 and osteoclast-marker expression following ACLY knockdown. ACLY-dependent H3 acetylation therefore links citrate metabolism to Rac1 transcription and the cytoskeletal machinery required for osteoclast differentiation and resorptive activity [3]. Consistent with these findings, pharmacological ACLY inhibition reduces osteoclast abundance and attenuates ovariectomy-associated bone loss in vivo. Because citrate-derived acetyl-CoA also supports osteogenic chromatin in osteoblast-lineage cells, ACLY represents a shared metabolic node whose effects depend on the lineage and transcriptional program engaged.

Acetate-sensitive chromatin regulation provides a distinct perspective on early osteoclast differentiation. Genome-wide profiling shows that RANKL initially reduces chromatin accessibility and H3K27ac, particularly during the early phase of differentiation. Acetate supplementation restores H3K27ac and chromatin accessibility at IRF8-associated regulatory regions and supports an IRF8-associated anti-osteoclastogenic transcriptional program, suppressing osteoclastogenesis [7]. This response contrasts with the pro-resorptive ACLY–GCN5–Rac1 pathway, illustrating how acetylation-associated regulation varies with differentiation stage and genomic context. Although the study links acetate exposure to changes in histone acetylation and chromatin accessibility, direct incorporation of acetate-derived carbon into histone acetyl groups was not established.

In mature osteoclasts, Rheb1-dependent mitochondrial respiration supports resorptive function through a distinct acetyl-CoA-associated mechanism. Osteoclast-specific Rheb1 deletion reduces oxidative phosphorylation and cellular acetyl-CoA, lowers cathepsin K (CTSK) abundance, and impairs collagen degradation. Acetate or citrate supplementation partially restores CTSK expression, whereas inhibition of ACSS2 or ACLY also reduces CTSK [8]. Notably, Rheb1 loss enhances multinucleated osteoclast formation ex vivo despite impairing CTSK-dependent resorption. High-dose acetate likewise increases global histone H3 acetylation while reducing CTSK expression, demonstrating that bulk histone acetylation does not necessarily predict the expression of a specific resorptive effector. Together, these studies show that acetyl-CoA metabolism supports distinct transcriptional and functional programs during osteoclast differentiation and maturation, with outcomes determined by the regulatory loci and cellular processes involved.

### 2.2. Citrate-Dependent Mitochondrial–Nuclear Communication During Osteogenic Differentiation

Acetyl-CoA cannot cross the inner mitochondrial membrane directly. Citrate export through the mitochondrial citrate carrier (CiC), encoded by SLC25A1, provides a major route for transferring mitochondrial acetyl units to the nucleocytosolic compartment, where ACLY regenerates acetyl-CoA for acetylation and other biochemical reactions. This citrate–CiC–ACLY pathway connects mitochondrial carbon metabolism with the acetyl-CoA available for chromatin regulation.

Studies in human adipose-derived MSCs have linked citrate transport to osteogenic differentiation and mitochondrial–nuclear organization. Osteogenic induction increases mitochondrial volume and the number and volume of mitochondria–nucleus contact sites, whereas inhibition of citrate transport impairs osteogenic gene expression and mineralization [9]. Citrate transport also influences multiple chromatin-associated pathways. Histone H3K9 acetylation increases during osteogenesis and declines when citrate transport is inhibited, while citrate-derived α-ketoglutarate promotes the removal of the repressive H3K9me3 mark. However, changes in these marks do not uniformly predict osteogenic outcomes: ACLY inhibition reduces H3K9ac while increasing osteogenic potential in the same model. The more direct rescue evidence supports a citrate–α-ketoglutarate–H3K9me3 pathway, indicating that mitochondrial citrate can influence osteogenic chromatin through distinct metabolite-dependent mechanisms.

Acetyl-CoA compartmentalization becomes particularly important during aging. Aged bone marrow MSCs accumulate total cellular acetyl-CoA despite reduced histone acetylation and chromatin accessibility at osteogenic regulatory regions. Increased mitochondrial-derived vesicle–lysosomal degradation lowers CiC abundance, restricting the transfer of mitochondrial carbon to the nucleocytosolic compartment. CiC re-expression restores acetyl-CoA export, histone acetylation, and osteogenic differentiation, while short-term acetate supplementation restores H3 acetylation and shifts the H3ac landscape toward an osteogenic state. Consistent with these findings, human bone marrow-derived MSCs from older osteoporotic donors show lower CiC abundance and histone H3 acetylation than cells from younger donors [5].

Exposure to atmospheric oxygen (21% O_2_) produces a related defect through a different upstream mechanism. Compared with culture at 2% O_2_, high-oxygen exposure increases total cellular acetyl-CoA but reduces CiC transport activity, promotes mitochondrial acetyl-CoA accumulation, decreases chromatin accessibility, and redistributes H3K27ac away from osteogenic promoters and enhancers [4]. Unlike aging, which reduces CiC abundance, high-oxygen exposure primarily affects transporter activity. Both conditions therefore impair citrate-dependent carbon transfer and uncouple total cellular acetyl-CoA abundance from the acetyl-CoA available for osteogenic chromatin regulation [4,5].

The functional consequences of acetyl-CoA availability also depend on differentiation stage. During early Wnt3a-induced lineage specification, reduced nuclear acetyl-CoA and global histone acetylation contribute to repression of the alternative adipogenic program [6]. By contrast, maintaining osteogenic chromatin in aged or oxygen-stressed MSCs requires sufficient nucleocytosolic acetyl-CoA availability [4,5]. Acetyl-CoA therefore does not exert a uniform effect on osteogenesis; its consequences depend on the metabolic environment, differentiation state, and transcriptional program being regulated.

Acetyl-CoA metabolism also influences osteogenic transcription through non-histone protein acetylation. In C3H10T1/2 mesenchymal progenitor cells, inhibition of oxidative metabolism suppresses osteogenic differentiation despite compensatory glycolysis that maintains cellular ATP, whereas enhanced mitochondrial oxidation increases β-catenin acetylation and transcriptional activity [10]. Pharmacological ACLY inhibition reduces β-catenin acetylation and attenuates the associated osteogenic response, linking citrate-dependent acetyl-CoA metabolism to the regulation of an osteogenic transcription factor. This pathway complements histone acetylation by connecting metabolic state directly to the activity of a non-histone transcriptional regulator.

### 2.3. Local Acetyl-CoA Regeneration and Acetate-Sensitive Chromatin Regulation

The acetyl-CoA available for histone acetylation depends on both its metabolic source and its intracellular regeneration. Skeletal-cell studies link changes in citrate metabolism and acetyl-CoA availability to histone acetylation, chromatin regulation, and cellular function.

In BMSCs exposed to oxidative stress, increased MMP-9 is accompanied by reduced citrate synthase abundance, lower citrate and acetyl-CoA levels, decreased H3K9ac, and impaired osteogenic differentiation. Pharmacological MMP-9 inhibition reverses these changes and improves bone parameters in ovariectomized mice [11]. In osteoarthritic chondrocytes, increased ACLY activity expands the nucleocytosolic acetyl-CoA pool and promotes H3K9ac and H3K27ac at inflammatory and matrix-catabolic genes, alongside acetylation of NF-κB p65 [12]. Periodontal ligament fibroblasts exhibit a related connection between acetyl-CoA availability and osteogenic chromatin: PPARγ depletion reduces H3K9ac and H3K27ac and impairs osteo/cementogenic differentiation, whereas exogenous acetyl-CoA partially restores the phenotype. Integrated RNA-seq and H3K27ac ChIP-seq further identify RUNX2, SULF2, RCAN2, and RGMA among PPARγ-associated active chromatin targets [13]. Together, these findings connect metabolic regulation of acetyl-CoA with distinct histone and non-histone acetylation programs across skeletal and related musculoskeletal cells.

Acetyl-CoA synthetase 2 (ACSS2) provides a mechanism for regenerating acetyl-CoA locally near chromatin. In non-skeletal systems, nuclear ACSS2 recaptures acetate released during histone deacetylation, sustaining acetyl-CoA availability and histone acetylation at transcriptionally active regions under nutrient or oxygen limitation [14,15,16]. In skeletal models, acetate and propionate promote early osteoblastic differentiation in MC3T3-E1 cells and primary osteoblasts [17], while acetate restores H3K27ac and chromatin accessibility during early osteoclast differentiation [7]. These findings establish acetate-responsive metabolic and chromatin programs in bone cells and provide a basis for investigating whether local ACSS2-dependent acetyl-CoA regeneration contributes to lineage-specific responses.

The gut–bone axis extends this regulation beyond individual cells. Microbial fermentation of dietary fiber supplies short-chain fatty acids (SCFAs), including acetate, propionate, and butyrate, that influence systemic bone metabolism. Lucas et al. showed that SCFA supplementation and high-fiber feeding increased bone mass and protected mice against ovariectomy- and inflammation-associated bone loss, primarily by suppressing osteoclast differentiation and resorption [18]. In osteoclast precursors, propionate and butyrate shifted metabolism toward glycolysis and away from oxidative phosphorylation, accompanied by reduced TRAF6 and NFATc1 expression. Short-term propionate supplementation in healthy human participants was also associated with lower circulating bone-resorption markers. These findings connect microbiota-derived metabolites with osteoclast metabolic programming and systemic bone turnover, complementing cell-intrinsic mechanisms of acetyl-CoA regulation.

### 2.4. Glucose Metabolism and Nucleocytosolic Acetyl-CoA Supply

Glucose metabolism is extensively remodeled during osteoblast differentiation. GLUT1-mediated glucose uptake supports RUNX2 stability and osteoblast differentiation, while RUNX2 promotes GLUT1 expression. GLUT1, GLUT3, and GLUT4 make distinct contributions to osteoblast proliferation and metabolism [19,20]. WNT–LRP5 and LGR4 signaling enhance aerobic glycolysis during osteogenic differentiation [21,22], while the relative contributions of glycolysis, mitochondrial oxidation, and biosynthetic pathways vary with differentiation state and signaling context [23,24,25].

Glucose utilization does not translate directly into nucleocytosolic acetyl-CoA availability, because glucose-derived carbon can enter lactate production, mitochondrial oxidation, or other biosynthetic pathways. Studies in non-skeletal systems demonstrate that glycolytic flux can influence acetyl-CoA abundance and locus-specific histone acetylation [26,27,28]. In osteoblast-lineage models, WNT signaling promotes aerobic glycolysis [21], whereas Wnt3a-induced commitment of bipotent progenitors reduces glucose-derived carbon entry into the tricarboxylic acid (TCA) cycle, accompanied by lower nuclear citrate, nuclear acetyl-CoA, and histone acetylation [6]. Thus, increased glycolytic activity can coexist with reduced nuclear acetyl-CoA availability, depending on how glucose-derived carbon is routed during lineage specification.

Osteoclast differentiation likewise involves extensive metabolic reprogramming. RANKL increases glycolytic activity and lactate production, while disruption of LDHA or PDK4 suppresses osteoclastogenesis [29,30,31]. ACOD1/itaconate and PKM2 further regulate glycolytic and inflammatory transcriptional programs through HIF-1α- and STAT3-associated mechanisms [32,33]. These pathways establish glucose metabolism as a determinant of osteoclast differentiation and inflammatory activity. Defining how glucose-derived carbon enters nucleocytosolic acetyl-CoA and specific histone acetyl groups will clarify the contribution of glucose metabolism to osteoclast chromatin regulation.

### 2.5. Fatty-Acid Oxidation as a Potential Acetyl-Group Source in Bone Cells

Long-chain fatty acids support osteoblast-lineage metabolism, with their contribution to skeletal function varying by sex, nutritional status, and physiological context. Osteoblast/osteocyte-specific disruption of Cpt2 impairs postnatal bone acquisition, particularly in female mice, while ATGL-dependent lipolysis mobilizes fatty acids from intracellular lipid droplets to sustain oxidative ATP production in matrix-producing osteoblasts under limited nutrient availability [34,35]. Wnt–LRP5 signaling promotes fatty-acid oxidation in osteoblasts [36], and mitochondrial β-oxidation of adipose-derived fatty acids contributes to PTH-induced bone formation [37]. These findings establish fatty-acid oxidation as an important component of skeletal bioenergetics.

Direct evidence that fatty-acid-derived carbon contributes to histone acetylation comes from a non-skeletal myeloid model. Using ^13^C-fatty-acid tracing, Wu et al. demonstrated incorporation of lipid-derived carbon into histone H3. Disruption of ACAT1 reduced this incorporation alongside H3 acetylation, STAT1-associated H3K9ac, and chromatin accessibility; carnitine/acylcarnitine transport also contributed to this pathway [38]. This mechanism provides a relevant precedent for osteoclast biology, given the monocyte/macrophage origin of osteoclasts. In skeletal models, disruption of long-chain fatty-acid oxidation impairs osteoclast formation, particularly in female mice, while CPT1A-dependent oxidation supports osteoclast-precursor fusion under inflammatory conditions [39,40].

Intracellular lipid partitioning further shapes these responses. DGAT1-dependent triglyceride synthesis limits palmitate-associated osteoclastogenesis, whereas loss of DGAT1 promotes this response [41]. FATP2-dependent fatty-acid uptake promotes osteoclastogenesis through changes in lipid metabolism and reactive oxygen species production. Sex-dependent differences in mitochondrial substrate oxidation further influence osteoclast metabolic responses [42,43]. These findings indicate that fatty-acid availability alone does not determine its biological effect; intracellular routing between oxidation and storage is also important. Whether fatty-acid-derived acetyl units enter the nucleocytosolic acetyl-CoA pool and contribute to histone acetylation in osteoblasts or osteoclasts remains to be established through isotope tracing in skeletal models (Table 1).

## 3. Lineage-Specific Chromatin Interpretation by Acetylation Machinery

Acetyl-CoA supplies the substrate for histone acetylation, but transcriptional specificity depends on where acetyltransferases are recruited and how they interact with lineage-specific transcription factors and chromatin environments. CBP/p300 illustrates how a shared acetyltransferase regulates osteogenic transcription through both histone and non-histone substrates. Osteoblast-specific loss of CBP or p300 reduces bone formation, bone mass, and mechanical strength, while pharmacological inhibition of their catalytic activity impairs osteoblast differentiation. At the chromatin level, CBP/p300-dependent H3K27ac supports β-catenin-associated transcription. In parallel, FOXO1 acetylation contributes to repression of *Stat1* [44]. These complementary mechanisms connect acetyltransferase activity to osteogenic gene regulation through distinct molecular substrates.

PCAF/KAT2B provides a more locus-resolved example of histone acetylation during osteogenic differentiation. BMP/SMAD signaling increases PCAF expression, and PCAF is recruited to the *BMP2*, *BMP4*, *BMPR1B*, and *RUNX2* promoters, where it promotes H3K9ac and transcription. PCAF depletion consequently reduces osteogenic gene expression, alkaline phosphatase (ALP) activity, mineralization, and MSC-mediated bone formation [45]. Because BMP/SMAD signaling induces PCAF while PCAF reinforces expression of multiple pathway components, this regulatory circuit supports sustained BMP-dependent osteogenic transcription.

MOF/KAT8 and GCN5/KAT2A extend this writer network through distinct chromatin targets. MOF expression and H4K16ac increase during osteogenic differentiation. MOF associates with *Runx2* and *Sp7* regulatory regions and interacts with RUNX2 and SP7 to support their transcriptional activity [46]. GCN5 promotes H3K9 acetylation and maintains BMSC stemness and osteogenic capacity [47]. Further evidence shows that GCN5 is recruited to the *Wnt1*, *Wnt6*, *Wnt10a*, and *Wnt10b* promoters, where increased H3K9ac supports Wnt/β-catenin-dependent osteogenesis [48]. In osteoclasts, the same acetyltransferase acts downstream of ACLY at Rac1 regulatory regions to support the cytoskeletal program required for resorption [3]. GCN5 therefore links acetyl-CoA metabolism to distinct transcriptional programs in bone-forming and bone-resorbing cells through lineage-specific genomic targets.

KAT6A extends locus-specific acetyltransferase activity into the osteoclast lineage. KAT6A depletion suppresses RANKL-induced osteoclast differentiation and attenuates ovariectomy-associated bone loss. KAT6A is enriched at the *Tet1* promoter, where its depletion reduces H3K9ac, RNA polymerase II recruitment, and *Tet1* transcription. TET1 subsequently associates with the *Trpv4* gene region, and overexpression of either TET1 or TRPV4 partially restores osteoclast differentiation following KAT6A depletion [49]. These findings place the KAT6A–TET1–TRPV4 axis downstream of promoter acetylation and link it to osteoclast differentiation. Together, these writer systems show that the skeletal effects of acetyltransferases depend on their genomic targets and interacting regulatory factors, rather than on enzyme abundance alone.

### 3.1. Transcription-Factor-Guided Targeting of Acetylation at Osteogenic Regulatory Regions

Lineage-associated transcription factors help determine where histone acetylation is established and maintained across osteogenic promoters and enhancers. ZNF462 provides a direct example of coordinated writer recruitment: it forms a complex with MOZ/KAT6A and RUNX2 at osteogenic regulatory regions, where the three factors support H3K9ac, H3K14ac, and H3K23ac. Osteoblast-specific Zfp462 deficiency consequently impairs bone formation and mechanical strength [50].

PLZF/ZBTB16 operates through a differentiation-dependent enhancer program in human MSCs. During osteogenic commitment, the ZBTB16 locus transitions from a Polycomb-associated, H3K27me3-enriched repressed state to one enriched in H3K27ac. PLZF subsequently occupies a subset of osteogenic enhancers and supports nearby gene activation. At a latent enhancer within the *ZBTB16* locus, activation coincides with PLZF, p300, and Mediator occupancy, as well as enhancer–promoter communication involving *NNMT* [51]. GATA4 provides a complementary example at the *RUNX2* locus. GATA4 binds the *RUNX2* promoter and an associated enhancer, maintaining H3K27ac, permissive chromatin marks, and local accessibility, although the acetyltransferase responsible for this H3K27ac state has not been identified [52]. Together, these studies show how transcription-factor occupancy and enhancer state direct acetylation toward specific components of the osteogenic program.

Locus-resolved analysis also reveals disease-associated chromatin changes that may be obscured by global measurements. In MSCs from patients with ankylosing spondylitis, global H3K27ac abundance differs little from that in control cells, yet H3K27ac profiling identifies SNP-adjacent super-enhancers associated with a distinct transcriptional network and enhanced osteogenic differentiation [53]. Time-dependent changes in histone acetylation during C3H10T1/2 osteogenesis further demonstrate that chromatin regulation evolves throughout differentiation [54]. These findings highlight the value of examining specific regulatory regions alongside global acetylation when characterizing osteogenic transcriptional programs.

Histone acetylation also belongs to a broader landscape of metabolite-dependent lysine acylation. Histone lactylation, which is associated with lactate metabolism rather than acetyl-CoA-dependent acetyl transfer, has been linked to osteoblast differentiation [55]. In MC3T3-E1 osteoblast-lineage cells, Nian et al. found that LDHA expression, lactate accumulation, and H3K18 lactylation increased during osteogenic differentiation. LDHA depletion reduced H3K18 lactylation at the *JunB* promoter, *JunB* expression, and osteogenic differentiation, whereas lactate supplementation restored the promoter-associated lactylation response [56]. Complementary work in BMP-2-induced C2C12 cells linked p300 perturbation to changes in histone lactylation and osteogenic differentiation [57]. These findings identify lactylation as a related metabolic–chromatin mechanism in skeletal-lineage models. Although acetylation and lactylation can involve overlapping regulatory machinery, they represent distinct histone modifications and should be interpreted according to their respective metabolic pathway.

### 3.2. Acetyl-Lysine Readers and Acetylation-Dependent Chromatin Processing

Histone acetylation influences chromatin regulation not only through changes in local chromatin state but also through the recruitment of acetyl-lysine readers and subsequent chromatin-processing events. Osteoclastogenesis provides a distinctive example in which acetylation precedes histone proteolysis. CBP/p300-dependent H3K18 acetylation promotes MMP-9-mediated cleavage of the H3 N-terminal tail near the transcription start sites of selected osteoclastogenic genes. Disruption of either H3K18 acetylation or MMP-9 activity impairs gene transcription and osteoclast differentiation [58]. This process also depends on the surrounding histone-modification state: G9a-dependent H3K27me1 facilitates MMP-9 localization and H3-tail cleavage at osteoclastogenic loci [59]. H3 clipping thus links histone acetylation to proteolytic chromatin remodeling within a combinatorial modification environment.

This pathway can be targeted at multiple steps. The tetracycline analogs tigecycline and minocycline inhibit MMP-9-mediated H3-tail cleavage and suppress osteoclast differentiation [60]. Cell-penetrating H3-tail peptides have also been shown to enter the nucleus and competitively interfere with p300-dependent H3K18 acetylation and MMP-9-mediated proteolysis, reducing osteoclastogenic gene expression and multinucleated osteoclast formation [61]. These findings identify H3 clipping as a potential intervention point in osteoclast chromatin regulation.

BET proteins recognize acetylated chromatin and connect histone marks with transcriptional regulators. In osteoclast precursors, I-BET151 suppresses RANKL-induced MYC expression and MYC recruitment to the *NFATC1* promoter. This response is accompanied by reduced CBP recruitment, histone H4 acetylation, and *NFATC1* transcription, ultimately suppressing osteoclast differentiation and pathological bone resorption [62]. Osteoblast responses vary with the mode of BET perturbation. BRD4 depletion impairs osteoblast differentiation and reduces its occupancy at osteoblast-associated regulatory regions in one mode [63]. In another study, BRD4-specific RNA interference increases mineralization, whereas pan-BET inhibition suppresses osteoblast differentiation [64]. These contrasting findings indicate that the skeletal effects of BET targeting depend on inhibitor selectivity, experimental model, and differentiation endpoint.

Histone acetylation also functions within a combinatorial modification landscape. During osteoclastogenesis, SETD2-dependent activation of Wnt5a involves increased H3K36me3 and reduced repressive H3K27me3. SETD2-associated NF-κB activity also promotes p300/PCAF recruitment and the accumulation of H3K9ac and H4K8ac around the Wnt5a transcription start region [65]. This locus illustrates how histone methylation, acetyltransferase recruitment, and acetylation cooperate to regulate a specific transcriptional program. Acetyl-lysine recognition and downstream chromatin processing therefore operate within a broader regulatory environment in which combinations of histone marks help determine transcriptional outcomes.

### 3.3. Deacetylases: Catalytic Activity, Complex Composition and Intracellular Localization

The effects of deacetylases in bone depend on their substrates, interacting proteins, and cellular context. In rat BMSCs, HDAC8 suppresses osteogenic differentiation by reducing H3K9ac and inhibiting RUNX2 transcriptional activity. HDAC8 knockdown increases H3K9ac, osteogenic gene expression, and mineralization [66]. Broad-spectrum HDAC inhibition produces a more complex response. In committed MC3T3 osteoblasts, transient treatment with suberoylanilide hydroxamic acid increases genome-wide histone H4 acetylation and promotes terminal differentiation and matrix mineralization, while reducing insulin receptor–Akt–FOXO1 phosphorylation [67]. These parallel chromatin and signaling responses illustrate why the skeletal effects of HDAC inhibition cannot be attributed to histone hyperacetylation alone.

HDAC3 illustrates how substrate selection and protein-complex composition shape deacetylase function. Conditional *Hdac3* deletion in Ctsk-expressing cells increases NF-κB p65 K310 acetylation, enhances RANKL responsiveness and osteoclast resorption, and reduces cortical and trabecular bone mass. The deletion also reduces osteoclast production of sphingosine-1-phosphate (S1P) and weakens the ability of osteoclast-conditioned medium to support osteoblast mineralization, implicating HDAC3 in both osteoclast-intrinsic activity and osteoclast-to-osteoblast coupling [68]. By contrast, shRNA-mediated HDAC3 suppression inhibits osteoclast differentiation [69]. This difference suggests that the consequences of HDAC3 perturbation depend on the extent and timing of depletion and the experimental model.

Mechanistic evidence further explains the context-dependent activity of HDAC3. RANK signaling promotes RNA-dependent assembly of an NCoR/HDAC3/PGC1β complex. Within this complex, HDAC3 deacetylates and activates PGC1β while exhibiting reduced activity toward histone H3. The complex consequently acts as a co-activator of AP-1- and NF-κB-dependent transcription [70]. HDAC3 therefore regulates osteoclast gene expression through both non-histone substrate modification and context-dependent chromatin interactions, rather than through a uniform increase or decrease in histone acetylation.

Class IIa HDACs, including HDAC4, HDAC5, HDAC7, and HDAC9, have weak intrinsic activity toward canonical acetyl-lysine substrates [71]. Their skeletal functions rely heavily on transcription-factor interactions, corepressor complexes, and regulated nuclear–cytoplasmic trafficking. HDAC7 interacts with MITF to suppress osteoclast differentiation through a mechanism partly independent of its deacetylase activity [69], whereas HDAC9 restrains osteoclastogenesis through reciprocal regulation with PPARγ/RANKL signaling [72]. Perturbation of HDAC4, HDAC5, HDAC9, HDAC10, or HDAC11 produces distinct effects on osteoclast maturation and matrix demineralization, supporting non-redundant functions among HDAC isoforms [73]. Phosphorylation-dependent trafficking provides an additional layer of regulation: HDAC5 nuclear localization changes independently of its total protein abundance [74].

HDAC4 also regulates osteogenic chromatin. In human adipose-derived stem cells, HDAC4 depletion increases histone H3 acetylation at osteogenic regulatory regions, including the *SMAD4* locus, and enhances SMAD4 expression and osteogenic differentiation. Given the weak intrinsic catalytic activity of class IIa HDACs, these findings are best interpreted as HDAC4-associated chromatin repression rather than direct evidence of H3 deacetylation by HDAC4 itself. The HDAC4-binding compound tasquinimod produces a similar osteogenic response, while sustained local delivery from a hydrogel enhances regeneration of critical-sized craniofacial defects [75]. This work links HDAC4-associated repression to a potential strategy for locally promoting skeletal regeneration.

SIRT6 demonstrates how the skeletal consequences of histone deacetylation depend on developmental stage and cellular lineage. Global Sirt6 deficiency produces low-turnover osteopenia, excessive H3K9ac at *Runx2*, *Sp7*, *Dkk1*, and *Opg* regulatory regions, impaired osteoblast differentiation, and reduced osteoclastogenesis [76]. In contrast, *Sirt6* deletion in mature osteoblasts and osteocytes lowers OPG expression and increases osteoclast formation without a comparable reduction in bone-formation parameters in vivo [77]. These distinct phenotypes reflect the different contributions of SIRT6 across skeletal lineages and developmental stages, including its effects on osteoblast differentiation and osteocyte-derived control of osteoclastogenesis.

SIRT1 provides a more direct link between NAD^+^-dependent deacetylation and osteocyte regulation of bone formation. SIRT1 is recruited to the Sost promoter, where it reduces H3K9ac and represses sclerostin expression [78]. Several upstream pathways converge on this regulatory axis. CK2-dependent phosphorylation of USP4 stabilizes SIRT1 by limiting ubiquitin-dependent degradation, PHD2/HIF-1α oxygen sensing enhances SIRT1-dependent deacetylation at the Sost promoter, and TSC1–mTORC1 signaling regulates Sirt1 expression in osteocytes [79,80,81]. Through these mechanisms, metabolic, oxygen-sensing, and intracellular signaling pathways converge on osteocyte-derived inhibition of Wnt signaling, linking chromatin regulation to osteoblast activity.

SIRT3 and SIRT2 extend deacetylase function beyond nuclear histones to mitochondrial and metabolic substrates. In osteoblasts, SIRT3-dependent deacetylation activates SOD2, limits mitochondrial oxidative stress, and supports differentiation [82]. SIRT3–SOD2 signaling also restrains RANKL-induced osteoclastogenesis by reducing mitochondrial reactive oxygen species (ROS) [83,84]. However, *Sirt3* deletion in aged or ovariectomized mice reduces osteoclast mitochondrial respiration, multinucleation, and resorptive activity, thereby attenuating bone loss under these conditions [85]. The effects of SIRT3 on osteoclast function therefore vary with age, estrogen status, and metabolic state. SIRT2 provides another example of metabolic regulation through non-histone deacetylation: in periodontal macrophages, SIRT2-mediated CPT1A deacetylation regulates fatty-acid metabolism [86]. Such pathways influence the metabolic environment in which chromatin regulation operates, while remaining mechanistically distinct from direct histone deacetylation.

Collectively, these findings show that deacetylases regulate skeletal remodeling through multiple mechanisms, including histone modification, non-histone substrate deacetylation, transcriptional complex assembly, and intracellular trafficking. Their effects on bone formation and resorption depend on the substrates engaged, the skeletal lineage involved, and the physiological context.

## 4. Mechanical Regulation of Chromatin in Osteogenic Cells and Osteocytes

Mechanical loading regulates skeletal-cell transcription through chromatin remodeling, deacetylase trafficking, and metabolic adaptation. In human bone marrow-derived mesenchymal stem/stromal cells (BMSCs), cyclic mechanical stretch reduces HDAC1 expression and increases histone H3 acetylation at the JAG1 promoter, activating JAG1–Notch signaling and enhancing osteogenic differentiation, alkaline phosphatase (ALP) activity, and mineralization. Consistent with this response, hindlimb unloading increases Hdac1 expression and suppresses Jag1–Notch signaling in vivo, while HDAC1 inhibition partially rescues unloading-associated bone loss [87]. The HDAC1–JAG1 axis thus links mechanical input to promoter acetylation and osteogenic adaptation.

In osteocytes, class IIa HDACs translate mechanical and hormonal signals into transcriptional control of Sost, which encodes the Wnt inhibitor sclerostin. HDAC5 associates with MEF2C at the +45 kb Sost enhancer and restrains its activity. Loss of HDAC5 increases MEF2C occupancy and H3K27ac while reducing recruitment of the NCoR/HDAC3 corepressor complex [88]. In contrast, class I HDAC activity supports full Sost transcription, indicating distinct roles for class I and class IIa HDACs in osteocyte regulation [88,89]. Osteocyte-specific Mef2c deletion reduces *Sost* expression and increases bone mass but also increases *Opg* expression and suppresses bone resorption through effects not reproduced by partial *Sost* deficiency [90]. MEF2C therefore coordinates multiple osteocyte-derived signals that influence both bone formation and resorption.

Focal adhesion kinase (FAK) connects cell–matrix force sensing with HDAC5 localization. Under adhesive conditions, FAK phosphorylates HDAC5 at Y642, regulating its subcellular distribution. Fluid-flow shear stress reduces HDAC5 Y642 phosphorylation and promotes nuclear accumulation of class IIa HDACs, leading to Sost suppression [91]. This pathway links mechanical sensing at the cell–matrix interface to the transcriptional control of osteocyte-derived signals that regulate bone formation.

Mechanical loading also remodels active chromatin beyond canonical bone-remodeling pathways. Transcriptomic and H3K27ac profiling associate loading with NRF2 stabilization and nuclear accumulation, increased H3K27ac at NRF2 motif-containing regulatory regions, and activation of antioxidant and mechanosensitive genes, including *Gsta2* and *Ptgs2* [92]. Pharmacological NRF2 stabilization does not fully reproduce the mechanically induced transcriptional response, suggesting that NRF2 operates within a broader loading-responsive regulatory network. These findings extend the chromatin response to mechanical stimulation from SOST-mediated control of bone formation to antioxidant defense and cellular adaptation.

Osteocyte metabolism provides a complementary dimension of mechanical adaptation. Parathyroid hormone (PTH) and the HDAC inhibitor Scriptaid both increase osteocyte respiration and suppress *Sost* expression, but their effects on chromatin regulation and metabolism are not identical. Scriptaid-induced *Sost* repression depends on HDAC5 nuclear accumulation and MEF2C regulation, whereas changes in metabolic gene expression and glucose utilization persist after disruption of HDAC4/5 signaling [93]. Mature osteocytes also display substrate flexibility: glycolytic restriction increases PPARδ- and CPT1A-associated fatty-acid oxidation, while mechanical loading increases *Pparδ* and *Cpt1a* expression and is accompanied by acylcarnitine accumulation in cortical bone [94]. These findings establish metabolic flexibility as a component of osteocyte adaptation to mechanical and hormonal stimuli. An important next step is to determine how changes in substrate utilization influence nucleocytosolic acetyl-CoA availability and histone acetylation at regulatory regions controlling *Sost*, antioxidant responses, and other osteocyte functions.

## 5. Acetylation-Dependent Regulation Within the Bone Remodeling Unit

**Figure 2:**
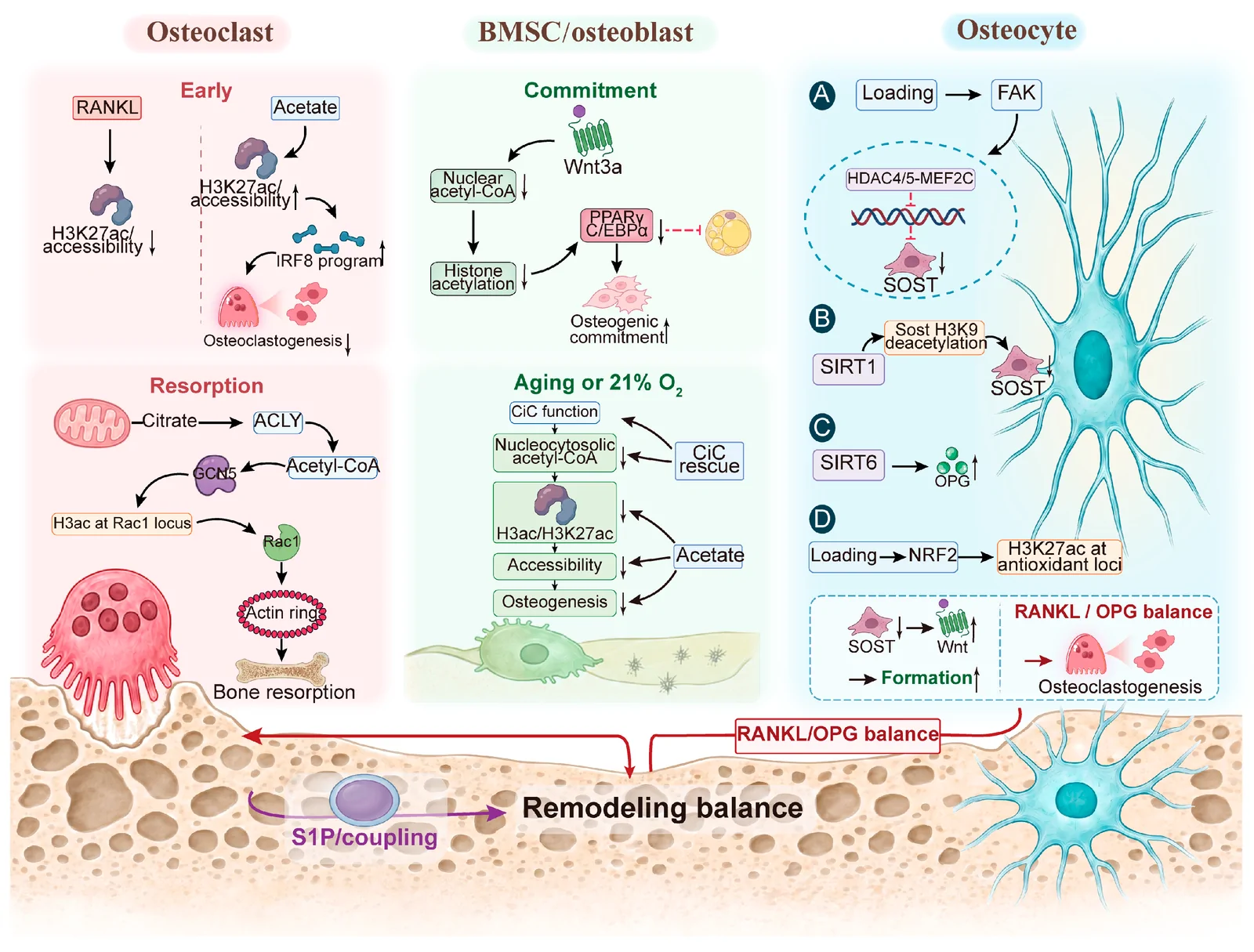
Lineage-, stage-, and locus-specific regulation of acetylation-associated programs across the bone-remodeling unit. The figure summarizes acetylation-associated regulatory programs in osteoclasts, BMSC/osteoblast-lineage cells, and osteocytes. In osteoclasts, RANKL-induced early differentiation is accompanied by reduced H3K27ac and chromatin accessibility, whereas acetate restores these features and promotes an IRF8-associated anti-osteoclast transcriptional program. During resorptive activity, citrate-derived acetyl-CoA supports ACLY–GCN5-associated H3 acetylation at Rac1 regulatory regions and the Rac1-dependent cytoskeletal program. In BMSC/osteoblast-lineage cells, Wnt3a reduces nuclear acetyl-CoA and global histone acetylation during early lineage commitment, suppressing PPARγ/C/EBPα-associated adipogenic programming and favoring osteogenic commitment. Aging or exposure to 21% O_2_ impairs CiC-dependent carbon transfer, reducing nucleocytosolic acetyl-CoA, histone acetylation, chromatin accessibility, and osteogenesis; CiC restoration or acetate supplementation partially reverses these changes. The osteocyte region contains four regulatory modules: (A) mechanical loading-associated FAK signaling and class IIa HDAC trafficking, with HDAC5–MEF2C-associated repression of Sost; (B) SIRT1-associated H3K9 deacetylation at the Sost promoter; (C) SIRT6-associated maintenance of OPG expression; and (D) loading-associated NRF2 activation accompanied by H3K27ac remodeling at antioxidant-response loci. At the remodeling-unit level, osteocyte-derived SOST/Wnt and RANKL/OPG signals, together with osteoclast-derived coupling factors such as sphingosine-1-phosphate (S1P), coordinate bone formation and resorption. The figure integrates experimentally supported skeletal mechanisms across lineages, while a direct metabolic acetyl-CoA–histone acetylation connection has not been established for every module.

Metabolic and chromatin regulatory mechanisms influence skeletal remodeling not only through cell-autonomous effects but also through communication among osteoblasts, osteoclasts, and osteocytes. Osteocyte-derived RANKL/OPG and SOST/Wnt signals, osteoclast-to-osteoblast coupling factors, and chromatin regulators shared across skeletal lineages connect intracellular acetylation programs with the balance between bone formation and resorption. This section integrates these mechanisms at the remodeling-unit level (Figure 2), providing a framework for understanding how aging, inflammation, estrogen deficiency, and metabolic stress disrupt coordinated skeletal remodeling.

### 5.1. Osteocyte Chromatin Coordinates RANKL/OPG and SOST/Wnt Signaling

Osteocytes coordinate bone remodeling through secreted signals that regulate both osteoclastogenesis and osteoblast activity. Myeloma-associated CIITA provides a direct example of how an osteocyte chromatin program can affect both processes. CIITA increases H3K14ac at the *Tnfsf11* and *Sost* promoters, elevating RANKL and sclerostin expression and thereby promoting osteoclastogenesis while suppressing osteoblastogenesis [95]. Osteocyte-specific *Ciita* deletion reverses these changes in myeloma-bearing mice, reducing osteoclast-mediated bone erosion and restoring bone formation. This pathway links locus-specific histone acetylation to coordinated changes in resorption and formation through osteocyte-derived signals.

Other acetylation regulators preferentially control individual components of this signaling network. SIRT1 reduces H3K9ac at the Sost promoter and suppresses sclerostin expression, linking NAD^+^-dependent deacetylation to Wnt-dependent bone formation [78]. SIRT6 in mature osteoblasts and osteocytes maintains OPG production and restrains osteoclastogenesis [77]. Upstream signaling further modulates these outputs: PHD2/HIF-1α oxygen sensing and CK2–USP4 signaling regulate SIRT1-dependent repression of Sost, while TSC1–mTORC1 signaling also influences SIRT1 and sclerostin expression [79,80,81]. Notably, osteocyte-specific Tsc1 deletion reduces sclerostin but increases RANKL and osteoclast formation, demonstrating that the SOST/Wnt and RANKL/OPG pathways can respond in opposite directions rather than functioning as a fixed regulatory pair.

Mechanical signals intersect with the same osteocyte chromatin network. Mechanical loading suppresses Sost through MEF2C–HDAC5 regulation and FAK-dependent trafficking of HDAC4/5 [88,89,90,91]. Loading also promotes NRF2 activation and H3K27ac remodeling at antioxidant-response loci, extending osteocyte chromatin adaptation to oxidative-stress defense [92]. Together, these findings show that the skeletal consequences of osteocyte chromatin regulation depend on the relative expression of multiple secreted signals, rather than on a uniform increase or decrease in histone acetylation.

### 5.2. Acetylation Regulators Influence Osteoclast-to-Osteoblast Coupling

Osteoclast-to-osteoblast coupling provides a direct link between intracellular acetylation regulation and subsequent bone formation. Conditional Hdac3 deletion in Ctsk-expressing cells increases osteoclast responsiveness to RANKL while reducing sphingosine-1-phosphate (S1P) output. Conditioned medium from HDAC3-deficient osteoclasts also supports osteoblast mineralization less effectively [68]. HDAC3 therefore influences both osteoclast resorptive activity and the production of signals that support osteoblast function. These responses need not change in parallel: enhanced osteoclastogenesis can coexist with impaired capacity to promote the bone-forming phase of remodeling.

Human bone-biopsy transcriptomic analyses following denosumab treatment identified LIF, CREG2, CST3, CCBE1, and DPP4 as candidate factors associated with remodeling-related changes [96]. These clinical observations complement experimental evidence implicating S1P in osteoclast-to-osteoblast communication. Together, the findings highlight the importance of evaluating both resorptive activity and coupling output when assessing acetylation-dependent changes in osteoclasts, as these functions can respond differently to the same regulatory perturbation.

### 5.3. Shared Chromatin Regulators Generate Lineage- and Context-Dependent Remodeling Effects

Several chromatin regulators function in both bone-forming and bone-resorbing lineages, but their transcriptional effects depend on cellular context. GCN5 supports Wnt-associated osteogenic transcription in BMSCs and acts downstream of ACLY at *Rac1* regulatory regions in osteoclasts [3,48]. BET inhibition suppresses osteoclast differentiation, whereas osteoblast responses vary with the extent and specificity of BET perturbation [62,63,64]. Similarly, CBP/p300 catalytic activity supports H3K27ac-dependent osteogenic transcription, while pharmacological inhibition of CBP/p300 suppresses osteoclast differentiation [44,97]. These findings demonstrate that shared chromatin regulators can control distinct transcriptional programs across skeletal lineages.

The tissue-level response consequently depends on how these cell-autonomous effects interact with intercellular signaling. Perturbing a shared regulator may suppress an osteoclastogenic program while also altering osteoblast activity. Osteocyte-derived RANKL/OPG and SOST/Wnt signals, together with osteoclast-derived coupling factors, further shape the resulting remodeling response. The net skeletal consequence of altering acetylation machinery therefore reflects the combined effects on bone resorption, bone formation, and intercellular communication. This remodeling-unit perspective provides the basis for understanding how disease and metabolic stress disrupt coordinated skeletal function.

## 6. Disease and Stress Contexts Reshape Metabolic–Chromatin Regulation

Aging, inflammation, estrogen deficiency, and metabolic stress alter acetyl-CoA metabolism and chromatin regulation across skeletal cell lineages, disrupting the balance between bone formation and resorption. These effects arise through distinct mechanisms, including changes in mitochondrial carbon transfer, locus-specific histone acetylation, transcriptional regulator activity, and intercellular signaling. Their skeletal consequences depend on the affected lineage and the regulatory pathways engaged in each physiological or pathological context.

### 6.1. Aging Alters Acetyl-CoA Distribution and Osteogenic Chromatin

Aging disrupts osteogenic chromatin through changes in both acetyl-CoA compartmentalization and the machinery that directs histone acetylation. Aged MSCs accumulate total cellular acetyl-CoA despite reduced nucleocytosolic acetyl-CoA availability, histone acetylation, and chromatin accessibility at osteogenic promoters and enhancers. Age-associated loss of the mitochondrial citrate carrier (CiC) restricts citrate-dependent carbon transfer, whereas CiC restoration or short-term acetate supplementation recovers histone acetylation and osteogenic capacity [5]. Aging also reduces ZNF462 expression in murine and human bone cells [50]. ZNF462 cooperates with MOZ/KAT6A and RUNX2 to establish H3K9ac, H3K14ac, and H3K23ac at osteogenic regulatory regions, while age-associated loss of H2A.Z and H3K4me3 at the ZNF462 locus contributes to reduced expression. Genome-wide H3K27ac profiling further reveals age-related changes in BMSC chromatin, including reduced H3K27ac at the *Igf2bp2* locus [98]. Together, these findings implicate both impaired acetyl-CoA availability and altered targeting of chromatin regulators in age-related loss of osteogenic potential.

Aging also compromises mitochondrial function and metabolic flexibility in stromal cells and mature osteoblasts, accompanied by impaired substrate utilization, lipid accumulation, and activation of oxidative-stress programs [99]. These metabolic changes reshape the substrate environment supporting nucleocytosolic acetyl-CoA production and may compound the chromatin defects associated with CiC loss and altered acetylation at osteogenic regulatory loci. Age-related skeletal decline therefore reflects disruption of coordinated metabolic and chromatin regulation rather than a uniform reduction in histone acetylation.

### 6.2. Inflammation Reshapes Histone and Non-Histone Acetylation in the Skeletal Microenvironment

Inflammation alters acetylation through distinct mechanisms in osteogenic stromal cells and inflammatory cells within the skeletal microenvironment. In periodontal ligament stem cells from patients with periodontitis or cells exposed to inflammatory stimuli, GCN5 expression and occupancy at the DKK1 promoter decline alongside H3K9ac and H3K14ac, reducing DKK1 expression and impairing osteogenic differentiation [100]. Although reduced DKK1 relieves inhibition of Wnt/β-catenin signaling, it does not restore osteogenesis in the inflammatory environment, indicating that pathway activation alone cannot overcome the broader disruption of osteogenic regulation.

A different mechanism operates in rheumatoid synovial fibroblasts, where neutrophil extracellular traps activate ACLY and increase acetylation and nuclear accumulation of NF-κB p65. This response enhances inflammatory fibroblast activation, whereas pharmacological ACLY inhibition reduces synovial inflammation and bone erosion in arthritis models [101]. Inflammation can therefore suppress histone acetylation at osteogenic regulatory loci while promoting acetyl-CoA-associated non-histone acetylation in inflammatory signaling pathways. In inflammatory osteoclasts, defining how metabolic reprogramming affects locus-specific histone acetylation will help clarify the contribution of chromatin regulation to cytokine-driven bone resorption.

### 6.3. Estrogen Deficiency Reveals Lineage-Dependent Responses to Acetylation Control

Estrogen deficiency exposes distinct metabolic and chromatin requirements in bone-resorbing and bone-forming cells. In osteoclasts, ACLY inhibition reduces nucleocytosolic acetyl-CoA and histone H3 acetylation, suppresses differentiation and resorption, and attenuates ovariectomy-associated bone loss [3]. In BMSCs exposed to an ovariectomy-associated oxidative-stress environment, reduced citrate synthase activity is accompanied by lower citrate, acetyl-CoA, and H3K9ac, together with impaired osteogenic differentiation; restoration of citrate metabolism improves osteogenic and skeletal outcomes [11]. Thus, restricting ACLY-dependent acetyl-CoA production can suppress osteoclast activity, whereas restoring citrate-associated metabolism supports osteogenic function under oxidative stress.

CBP/p300 provides a further example of a shared acetylation regulator with distinct effects across skeletal lineages. Pharmacological inhibition of CBP/p300 catalytic activity suppresses osteoclastogenesis and protects against ovariectomy-induced bone loss [97]. Conversely, osteoblast-specific loss of CBP or p300 reduces bone formation and mechanical strength, while their acetyltransferase activity supports H3K27ac-dependent osteogenic transcription [44]. These findings highlight the challenge of targeting shared acetylation machinery: an intervention that limits excessive resorption may also compromise the chromatin programs required for bone formation.

Deacetylase-targeted interventions further illustrate how pharmacological selectivity shapes skeletal outcomes. The broad-spectrum HDAC inhibitor quisinostat reduces osteoclastogenesis and bone resorption while increasing bone-formation indices in ovariectomized mice [102]. By contrast, the HDAC1-selective inhibitor OCP-001 preferentially suppresses osteoclast differentiation through a Blimp-1/IRF8-associated program and preserves bone mass after ovariectomy [103]. SIRT1 activation provides another approach through osteocyte-mediated regulation of bone formation. The SIRT1 activator SRT3025 suppresses sclerostin expression and improves bone mass, microarchitecture, and biomechanical properties in ovariectomized mice [104], consistent with SIRT1-mediated repression of Sost in osteocytes. Together, these studies show that protection against estrogen-deficiency-induced bone loss can arise through distinct cellular mechanisms. Evaluating such interventions requires consideration of both resorption and formation, alongside their effects on osteocyte signaling and communication within the bone-remodeling unit.

### 6.4. Nutrient Excess and Diabetes Reshape Substrate Use and Chromatin Regulation

High-fat-diet models reveal changes in both chromatin state and intracellular lipid handling. Maternal high-fat diet alters H3K27ac and H3K27me3 profiles in fetal osteoblast-like cells and offspring bone, including increased H3K27ac at the osteoblast-inhibitory *Tnfaip3* and *Twist1* loci and increased H3K27me3 at genes such as *Pthlh* and *Col2a1* [105]. In adult high-fat-diet-fed mice, osteoprogenitor-specific Plin2 deletion reduces lipid-associated osteoblast dysfunction and preserves bone formation and microarchitecture through increased fatty-acid oxidation [106]. Together, these findings implicate both chromatin regulation and intracellular lipid partitioning in skeletal responses to nutrient excess.

Diabetic bone provides a complementary example in which altered carbon routing has been directly characterized. In male mice with type 2 diabetes, in vivo ^13^C-glucose tracing demonstrates reduced glycolysis and glucose entry into the tricarboxylic acid cycle in bone. Single-cell analyses further reveal heterogeneous metabolic defects among marrow stromal populations, while osteoblast-targeted activation of glycolysis prevents diabetic bone loss [107]. In type 1 diabetes models, increasing glucose uptake through osteoblast GLUT1 overexpression exacerbates skeletal defects, whereas PFKFB3-driven glycolytic activation preserves cortical and trabecular bone mass [108]. These findings establish that the intracellular fate of glucose, rather than glucose availability alone, is a key determinant of skeletal outcomes in diabetes. Whether altered glucose routing changes nucleocytosolic acetyl-CoA availability or directs glucose-derived carbon into specific histone acetyl groups remains to be determined.

An adipocyte-rich marrow environment provides another link between metabolic context and osteogenic chromatin. Osteoblasts derived from orchiectomized osteoporotic rats, as well as osteoblasts exposed to signals generated through osteoblast–adipocyte interactions, exhibit reduced histone H3 acetylation and impaired osteogenic differentiation [109]. Trichostatin A restores histone H3 acetylation, RUNX2 expression, and osteogenic activity in both settings, supporting a functional role for histone deacetylation in the osteogenic defect. Across nutrient excess, diabetes, and marrow adiposity, metabolic substrate handling and chromatin regulation emerge as complementary determinants of skeletal function.

### 6.5. Complementary Non-Histone Acetylation in the Metastatic Bone Microenvironment

Non-histone acetylation provides an additional route through which metabolite availability influences communication within pathological bone microenvironments. In breast cancer bone metastasis, osteolytic degradation of type I collagen releases hydroxyproline, which undergoes PRODH2-associated metabolism in tumor cells and increases intracellular acetyl-CoA. PRODH2 promotes the interaction between KAT7 and the transcription factor YY1, enhancing YY1 acetylation at K230, stabilizing YY1, and supporting *IL8* transcription. Tumor-derived IL-8 subsequently promotes osteoclast differentiation and reinforces osteolytic activity [110]. This pathway links matrix-derived metabolites to non-histone acetylation and paracrine signaling, illustrating how metabolic–acetylation regulation can influence bone destruction through mechanisms distinct from histone-centered regulation in skeletal cells.

## 7. Therapeutic Potential and Translational Challenges

Targeting metabolic–chromatin pathways offers opportunities to modify pathological bone remodeling, but therapeutic outcomes depend on which skeletal lineages are affected and how selectively a target can be modulated. Current strategies range from inhibiting acetyl-CoA production and chromatin regulators to manipulating treatment duration and drug delivery. The central challenge is to suppress excessive resorption or restore impaired formation without disrupting the coordinated functions of osteoblasts, osteoclasts, and osteocytes.

### 7.1. Targeting Acetyl-CoA Supply and Compartmentalization

ACLY and the mitochondrial citrate carrier (CiC) illustrate how manipulating acetyl-CoA metabolism can produce distinct effects across skeletal lineages. In osteoclasts, the ACLY inhibitor BMS-303141 reduces nucleocytosolic acetyl-CoA and histone H3 acetylation, suppresses osteoclast differentiation and resorption, and attenuates ovariectomy-associated bone loss [3]. Conversely, restoring CiC function or supplementing acetate improves osteogenic chromatin regulation and differentiation in aged or oxygen-stressed BMSCs [4,5]. These findings support two complementary strategies: limiting acetyl-CoA availability in pathological bone resorption and restoring its accessibility in metabolically compromised osteoblast-lineage cells.

Bempedoic acid, an approved lipid-lowering drug that inhibits ACLY, illustrates how tissue-dependent drug activation can improve pharmacological selectivity. It is converted to its active CoA derivative by very-long-chain acyl-CoA synthetase 1 (ACSVL1), whose expression favors hepatic activation [111]. This mechanism provides a useful precedent for targeting a broadly expressed metabolic enzyme through tissue-selective activation. However, its established lipid-lowering efficacy does not demonstrate therapeutic activity in bone. Skeletal applications will require evidence of drug exposure and target engagement in the relevant bone-cell populations.

ACSS2 offers another potential point of intervention by regenerating acetyl-CoA from acetate. In non-skeletal systems, nuclear ACSS2 supports histone acetylation by recycling acetate released during deacetylation [14,15,16]. In CD8 T cells, ACSS2-dependent acetate utilization can also compensate for ACLY loss and maintain histone acetylation and chromatin accessibility [112]. This metabolic flexibility highlights a potential limitation of inhibiting a single acetyl-CoA-generating enzyme. Future strategies targeting ACLY, CiC, or ACSS2 will benefit from assessing compensatory carbon-routing pathways alongside target engagement and skeletal outcomes.

### 7.2. Targeting Shared Chromatin Regulators

BET proteins and CBP/p300 illustrate the challenges of targeting chromatin regulators that function across skeletal lineages. BET inhibition suppresses osteoclastogenesis and pathological bone resorption, but osteoblast responses vary with the mode of perturbation: pan-BET inhibition can suppress osteoblast differentiation, whereas BRD4-specific depletion produces model-dependent effects [62,63,64]. CBP/p300 presents a related lineage-selectivity problem. The catalytic inhibitor A-485 suppresses osteoclastogenesis and attenuates ovariectomy-associated bone loss [97], while CBP/p300 acetyltransferase activity supports H3K27ac-dependent osteogenic transcription and bone formation [44]. Pharmacological selectivity for a molecular target therefore does not guarantee selectivity for a skeletal lineage.

Clinical experience also underscores the importance of evaluating skeletal outcomes during prolonged treatment. Valproate, a widely used antiepileptic drug with HDAC-inhibitory activity, has been associated with reduced bone mineral density [113]. A systematic review and meta-analysis of 19 studies reported lower bone mineral density among patients receiving valproate monotherapy, particularly with prolonged treatment [114]. Although this association cannot be attributed exclusively to HDAC inhibition, it provides a clinically relevant reason to monitor bone health when using systemic agents that affect acetylation-related pathways.

More focused chromatin interventions may offer alternative strategies. Tigecycline and minocycline inhibit MMP-9-dependent histone H3-tail cleavage and suppress osteoclast differentiation, while cell-penetrating H3-tail peptides interfere with p300-dependent H3K18 acetylation and subsequent proteolysis [60,61]. KAT6A depletion also suppresses the TET1–TRPV4 pathway and attenuates ovariectomy-associated bone loss [49]. Selective KAT6A/B inhibitors, including WM-8014 and WM-1119, provide pharmacological tools for further investigation [115]. Because KAT6A also participates in the ZNF462–RUNX2 complex that supports osteogenic transcription [50], its therapeutic potential will depend on whether pathological osteoclast activity can be modified without compromising bone formation. These approaches remain at the preclinical stage for skeletal applications.

### 7.3. Cell-Selective and Temporally Restricted Intervention Strategies

Restricting the duration or anatomical distribution of treatment offers a practical way to reduce unwanted effects on other skeletal lineages. Transient ex vivo epigenetic priming provides one approach: a 48 h pretreatment with the HDAC2/3-selective inhibitor MI192 enhances subsequent osteogenic differentiation and mineralization of human dental pulp stromal cells after drug withdrawal [116]. A related strategy uses extracellular vesicles (EVs) from trichostatin A-treated osteoblasts. These EVs enhance human BMSC osteogenic differentiation without requiring direct exposure of recipient cells to the drug [117]. Both approaches separate the initial epigenetic intervention from the subsequent regenerative response.

Anatomically localized delivery provides another form of selectivity. In human adipose-derived stem cells, HDAC4 perturbation increases histone acetylation at osteogenic regulatory regions, including SMAD4, and promotes osteogenesis. Tasquinimod reproduces this response, while sustained delivery through a GelMA–F127 hydrogel enhances regeneration of critical-sized cranial and mandibular defects in mice [75]. This approach confines exposure to the regenerative site, although local delivery does not itself confer molecular or locus specificity. Its applicability to systemic osteoporosis or other skeletal disorders will require separate evaluation because localized defect repair and prevention of generalized bone loss involve different biological and structural outcomes.

By contrast, systemic activation of SIRT1 with SRT3025 engages the osteocyte SIRT1–Sost pathway and improves bone mass, microarchitecture, and biomechanical properties in ovariectomized mice [78,104]. This provides a pharmacological example of targeting osteocyte-mediated regulation of bone formation, but systemic administration does not ensure osteocyte-selective exposure.

**Figure 3:**
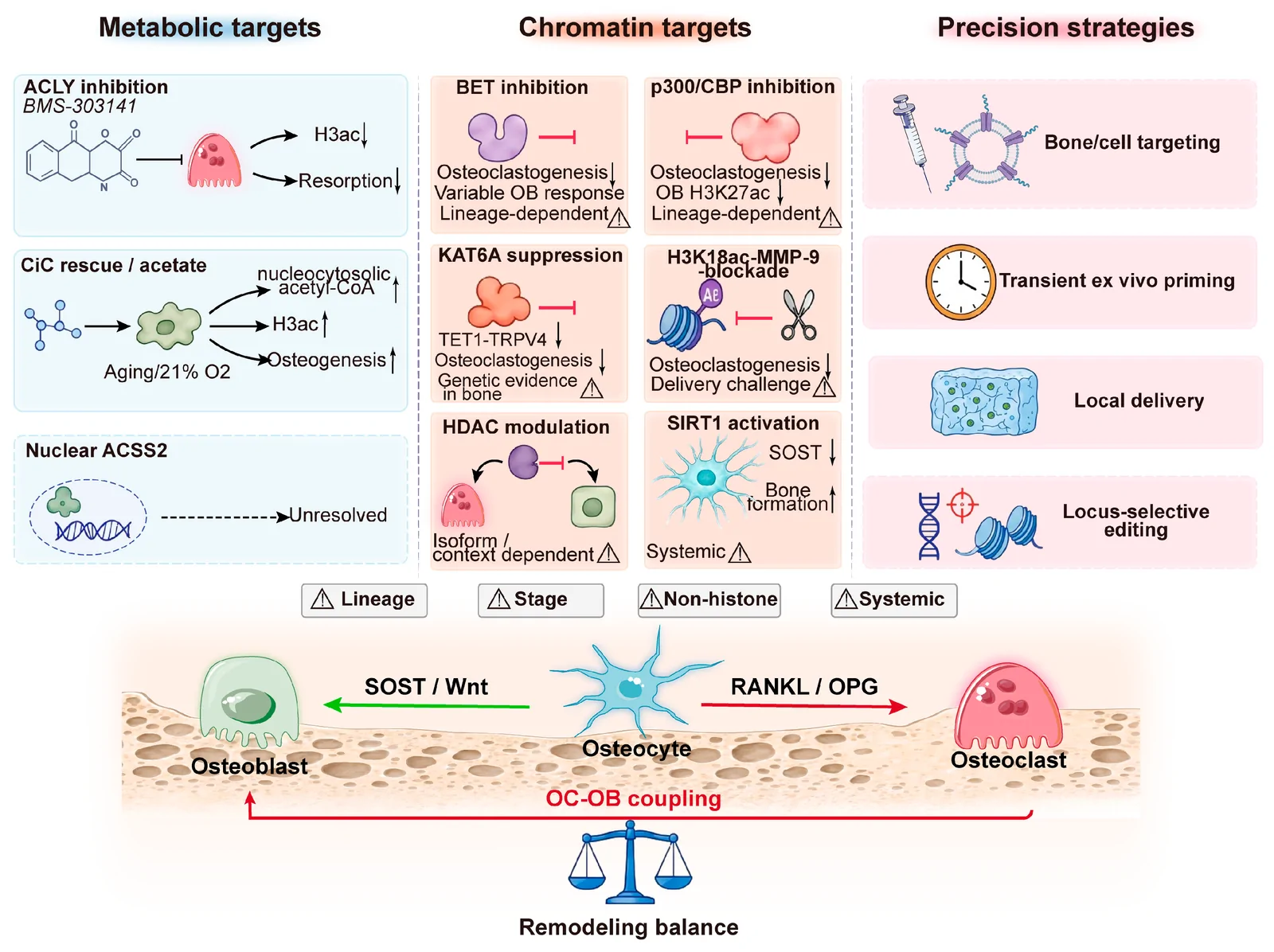
Preclinical evidence and prospective therapeutic strategies for targeting metabolic and acetylation pathways in bone. Therapeutic approaches are organized into three categories: metabolic targets, chromatin regulators, and strategies designed to improve spatial or temporal selectivity. Metabolic interventions include ACLY inhibition in osteoclasts, restoration of CiC-dependent acetyl-CoA availability in metabolically compromised BMSCs, and nuclear ACSS2-mediated acetate utilization, a candidate mechanism supported primarily by non-skeletal studies. Chromatin-directed approaches include BET and CBP/p300 inhibition, KAT6A modulation, interference with H3K18ac-associated MMP-9-dependent histone processing, HDAC modulation, and SIRT1 activation. Solid outlines denote approaches supported by experimental evidence in skeletal or bone-related models, whereas dashed outlines indicate candidate mechanisms or strategies whose skeletal application has not been established. Non-skeletal mechanistic evidence is distinguished where relevant. Transient ex vivo priming and local delivery illustrate approaches to limiting drug exposure, while bone-cell-targeted delivery and locus-selective epigenetic editing represent prospective strategies. The therapeutic potential of these approaches depends on their effects across skeletal lineages, their histone and non-histone substrates, and the balance between bone formation and resorption. The overall objective is to modify pathological bone remodeling while preserving osteoblast activity, osteocyte signaling, and osteoclast-to-osteoblast coupling.

Further translational development should prioritize pharmacokinetic and pharmacodynamic evidence of target engagement in the relevant skeletal compartments. Therapeutic efficacy should be evaluated through complementary measures of bone formation, resorption, microarchitecture, and mechanical competence, together with treatment durability and long-term skeletal safety. Integrating these outcomes with lineage-resolved metabolic and chromatin measurements will help identify interventions that modify pathological remodeling while preserving physiological bone function (Figure 3).

## 8. Research Priorities and Future Directions

### 8.1. Methodological Requirements for Establishing Metabolic Control of Histone Acetylation in Bone Cells

Establishing metabolic control of histone acetylation requires distinguishing total acetyl-CoA abundance, compartment-specific availability, and the metabolic origin of histone acetyl groups. Whole-cell acetyl-CoA measurements integrate multiple intracellular pools and cannot establish how much acetyl-CoA is available for nuclear acetylation reactions. Although nuclear and cytosolic acetyl-CoA can exchange through nuclear pores, differences in local production, consumption, and metabolic turnover may create functionally distinct environments. Subcellular fractionation introduces an additional technical challenge because metabolite redistribution, cross-contamination, and rapid turnover can distort compartment-specific estimates. Rapid sample quenching, validated fractionation procedures, and complementary measurements are therefore important for interpreting acetyl-CoA compartmentalization.

The resolution of histone acetylation measurements determines which mechanistic conclusions can be drawn. Immunoblotting and related bulk assays quantify global histone acetylation but cannot identify the promoters or enhancers responsible for a transcriptional phenotype. ChIP-seq and CUT&RUN, combined with chromatin-accessibility profiling, can identify regulatory regions associated with altered acetylation and transcription. However, these approaches do not by themselves establish that a particular acetylation event causes the associated gene-expression or skeletal phenotype. Stable-isotope tracing provides complementary information about metabolic carbon routing, whereas mass-spectrometric analysis of labeled histone acetyl peptides is required to establish the incorporation of substrate-derived carbon into histone acetyl groups.

Metabolite supplementation and rescue experiments also require careful interpretation. Acetate concentration, treatment duration, and the physiological relevance of substrate exposure can influence both metabolic responses and chromatin regulation. High-dose acetate, for example, increases global H3 acetylation while reducing CTSK expression in osteoclasts, demonstrating that bulk histone acetylation does not necessarily predict a corresponding functional response [8]. Dose–response experiments, appropriate controls, isotope-resolved tracing, and targeted perturbation of candidate metabolic pathways can help distinguish substrate availability from the specific mechanisms responsible for a cellular phenotype.

Together, these methodological considerations support an integrated experimental strategy combining compartment-resolved metabolite measurements, isotope tracing, locus-specific chromatin analysis, and functional perturbation. Each approach addresses a distinct step in the relationship between metabolic carbon, histone acetylation, transcription, and skeletal-cell function.

### 8.2. Resolving Carbon Sources, Chromatin Targets, and Remodeling-Unit Consequences

A major research priority is to determine which metabolic substrates supply histone acetyl groups in specific skeletal-cell states. Stable-isotope tracing with labeled glucose, fatty acids, acetate, and selected amino acids should be combined with quantitative acetyl-CoA measurements and mass-spectrometric analysis of histone acetyl peptides [118]. Evidence that fatty-acid-derived carbon can enter histone H3 in myeloid cells provides a useful experimental framework for osteoclast studies [38], while CiC studies in BMSCs demonstrate why total cellular acetyl-CoA alone cannot resolve the metabolic supply available to chromatin [4,5]. Applying these approaches across differentiation, aging, altered oxygen tension, and metabolic stress would clarify how carbon-source utilization changes the acetylation landscape in bone cells.

Osteocytes provide a particularly informative setting for integrating metabolic and chromatin regulation. Combining isotope-labeled substrate tracing with compartment-resolved acetyl-CoA measurements, histone acetyl-peptide analysis, and locus-specific chromatin profiling during mechanical stimulation could establish whether altered glucose or fatty-acid utilization contributes to acetylation at Sost-associated or mechanosensitive regulatory regions. Linking these measurements to targeted metabolic perturbations and osteocyte functional readouts would help determine how substrate utilization influences osteocyte-derived signals that regulate bone formation and resorption.

A second priority is to resolve the genomic locations and molecular substrates through which metabolic changes influence transcription. Global H3 or H4 acetylation does not necessarily reflect the regulatory regions that determine osteogenic or osteoclastic gene expression. CUT&RUN or ChIP-seq combined with chromatin-accessibility profiling can identify promoters and enhancers that respond to metabolic perturbation. Complementary acetyl-proteomics and site-directed mutagenesis can distinguish histone acetylation from modifications of transcription factors and metabolic enzymes. Locus-selective epigenome editing offers a further means of testing whether acetylation at individual regulatory elements contributes causally to transcriptional and skeletal outcomes, although this approach remains largely unexplored in skeletal cells [119].

Cellular heterogeneity and intercellular communication represent the next level of mechanistic resolution. Single-cell transcriptomic and chromatin-accessibility profiling has identified age- and differentiation-stage-associated regulatory states within the osteoclast lineage [120,121]. Extending these approaches to metabolic flux and histone acetylation would help identify cell states in which particular metabolic–chromatin pathways are most active. Spatial multi-omics could further resolve these relationships within the intact remodeling unit while preserving the anatomical organization of bone-forming, bone-resorbing, and stromal populations [122]. Combined with inducible lineage-restricted genetics and measurements of RANKL/OPG, SOST/Wnt signaling, osteoclast-derived coupling factors, and dynamic bone formation and resorption, these approaches could establish how cell-autonomous chromatin changes propagate through the remodeling unit.

Future studies should also account systematically for sex, chronological and developmental age, hormonal status, nutritional conditions, skeletal site, and experimental model. Comparisons across sexes, age groups, cortical and trabecular compartments, and primary-cell and in vivo models will help determine whether a metabolic–chromatin pathway has consistent skeletal effects or acts preferentially in a particular context. Oxygen tension and substrate availability are especially relevant when interpreting acetyl-CoA compartmentalization in cultured skeletal cells. Integrating these variables with isotope-resolved carbon flux, compartment-specific metabolite measurements, locus-resolved histone acetylation, and remodeling-unit phenotyping will improve the identification of mechanisms that are reproducible and therapeutically relevant.

## 9. Conclusions

Histone acetylation in bone is governed by the intersection of metabolic carbon routing, acetyl-CoA compartmentalization, and locus-specific chromatin regulation. Total cellular acetyl-CoA does not reliably predict histone acetylation: mitochondrial carbon must reach the nucleocytosolic compartment, and acetyltransferases, acetyl-lysine readers, and deacetylases direct the resulting chromatin response toward specific regulatory regions. The functional consequences vary with skeletal lineage, differentiation stage, and genomic context, explaining why changes in acetyl-CoA availability can support lineage restriction during early differentiation yet impair osteogenic maintenance under aging or metabolic stress. Findings from non-skeletal systems further identify local acetate recycling and lipid-derived histone acetylation as potential metabolic routes whose contributions to skeletal chromatin remain to be established.

These cell-intrinsic mechanisms are integrated across the remodeling unit through osteocyte-derived RANKL/OPG and SOST/Wnt signals, osteoclast-derived coupling factors, and other intercellular outputs. Mechanical loading, aging, inflammation, estrogen deficiency, and metabolic disease alter different components of this network rather than producing a uniform increase or decrease in histone acetylation. Therapeutic strategies targeting acetyl-CoA metabolism or chromatin regulators must therefore account for skeletal lineage, subcellular compartment, anatomical site, and treatment duration. Integrating carbon-flux analysis with locus-specific chromatin profiling and remodeling-unit phenotyping provides a framework for explaining how metabolic states are translated into skeletal outcomes and for developing interventions that modify pathological bone remodeling while preserving physiological bone formation, resorption, and intercellular coupling.

## Figures and Tables

**Table 1:** Metabolic routing, acetyl-CoA accessibility, and chromatin regulation: an evidence framework for skeletal cells.

Pathway	Model/Lineage	Key Readout	Skeletal Effect	Evidence Boundary
ACLY–GCN5–Rac1	Mouse osteoclast precursors; OVX mice	Rac1-associated H3ac	ACLY inhibition reduces osteoclastogenesis and resorption	Skeletal chromatin link; histone-carbon tracing not shown
Citrate routing (CiC/ACLY; CS/MMP-9)	Mouse BMSCs; human BMSCs	Global H3ac/H3K9ac; H3K27ac and chromatin accessibility	CiC restoration or MMP-9 inhibition improves osteogenesis	Skeletal evidence; direct carbon transfer/locus-specific causality unresolved
Glucose routing/Wnt3a	Murine ST2 progenitors; diabetic mouse bone	Nuclear acetyl-CoA/H3ac; ^13^C-glucose flux	Wnt3a reduces nuclear acetyl-CoA/H3ac; osteoblast glycolysis protects diabetic bone	^13^C-glucose traces bone flux, not histone-acetyl incorporation
Acetate–IRF8/ACSS2	Mouse osteoclast precursors and osteoblasts; non-skeletal ACSS2 models	H3K27ac/accessibility at IRF8-related regions	Acetate suppresses early osteoclastogenesis and supports osteoblast differentiation	Skeletal acetate response; ACSS2-dependent route shown mainly outside bone
Rheb1–acetyl-CoA–CTSK	Osteoclast-specific Rheb1 knockout; ex vivo cultures	Global H3ac; CTSK; collagen degradation	Rheb1 loss impairs resorption despite increased multinucleation	Global H3ac does not resolve CTSK-locus regulation or predict resorption
Fatty-acid oxidation	Mouse skeletal models; myeloid cells for tracing	Skeletal FAO phenotypes; ^13^C-fatty-acid-labeled H3 in myeloid cells	FAO supports bone acquisition and context-dependent osteoclast function	Histone-carbon tracing shown in myeloid, not skeletal cells
β-catenin acetylation	Murine C3H10T1/2 progenitors	Non-histone β-catenin acetylation	Supports osteogenic transcription	Complementary non-histone mechanism

Abbreviations: ACLY, ATP-citrate lyase; ACSS2, acetyl-CoA synthetase 2; BMSC, bone-marrow mesenchymal stromal cell; CiC, mitochondrial citrate carrier; CS, citrate synthase; CTSK, cathepsin K; H3ac, histone H3 acetylation; H3K9ac/H3K27ac, H3 lysine 9/27 acetylation; OVX, ovariectomy.

## Data Availability

No new data were created or analyzed in this study. Data sharing is not applicable to this article.
